# Local environmental quality and heterogeneity in an OLG agent-based model with spatial externalities

**DOI:** 10.1007/s11403-022-00346-9

**Published:** 2022-02-03

**Authors:** Andrea Caravaggio, Mauro Sodini

**Affiliations:** 1 Department of Economics, University of Foggia, Foggia, Italy; 2 Department of Law, University of Naples Federico II, Naples, Italy; 3 Department of Finance, Faculty of Economics, Technical University of Ostrava, Ostrava, Czech Republic

**Keywords:** Agent-based models, Overlapping generations, Local environment, Environmental externalities, C63, D62, O13, Q2

## Abstract

Most of the theoretical contributions on the relationship between economy and environment assume the environment as a good distributed homogeneously among agents. The aim of this work is to relax this hypothesis and to consider that the environment can have a local character even if conditioned through externalities by the choices made at the global level. In this article, we adapt the classical framework introduced in John and Pecchenino (Econ J 104(427):1393–1410, 1994) to analyze the dynamic relationship between environment and economic process, and we propose an OLG agent-based model where each agent perceives her own level of environmental quality determined by her own decisions, and by the decisions of those living around her. Despite the attention devoted to local environmental aspects, network externalities (determined through the scheme of Moore neighborhoods) play a fundamental role in defining environmental dynamics and they may induce the emergence of cyclical dynamics. The occurrence of oscillations in the local environmental quality is partially mitigated by the presence of heterogeneity in individuals’ preferences. Finally, when a centralized planner is introduced, the dynamics converge to stationary values regardless of the assumption on heterogeneity of agents.

## Introduction

In recent years, environmental issues have assumed an increasing relevance both in economic research and in political agendas. From a theoretical point of view, modeling approaches to environmental issues are characterized by crucial differences. The literature that explored this topic in a dynamic setting offers a traditional view of the environmental problem solved by an infinitely lived benevolent social planner. Leaving aside the specific differences within these works, this approach is mainly characterized by a social planner with perfect knowledge about environmental dynamics aiming to optimally allocate resources between economic activity and environmental protection (see Van Der Ploeg and Withagen 1991). The result typically obtained in this strand of literature is the existence of a path on which the economy monotonically grows toward the long-term equilibrium and environmental quality increases accordingly or, alternatively, it exhibits an U-shaped evolution. However, even in the second case, after an initial phase of degrowth, the prediction of such models is the monotonic evolution of the environmental quality toward its stationary level or growth rate.^1^ Alongside this approach, another strand of research has focused on the study of the characteristics of economic systems when environmental issues are solved by a social planner, with a finite decision-making horizon. In this regard, some examples are represented by the articles of John and Pecchenino (1994), John et al. (1995) and Fodha and Seegmuller (2014). Though from a theoretical point of view there are important differences (the non-Pareto optimality of the trajectories), from a dynamic point of view, results are similar to those described so far. Indeed, in several works (such as John et al. 1995), the authors focus on identifying the stationary states of the system and analyzing how the levels of these equilibria are affected by the main parameters of the model. In other words, although models are dynamic, since the growth process leads the system closer and closer to stationary states, the analysis is restricted to these. Results dramatically change when a decentralized solution to environmental issues is considered. As shown in several works by Antoci and coauthors (see Antoci et al. 2007, 2019), the introduction of a representative agent, which unlike the social planner does not internalize the environmental problem, may produce dynamics that do not converge to any long-term equilibrium. In this case, economic variables oscillate forever even if no exogenous shock is introduced. The main message of these articles is that, due to the distortions they create in the allocation process, environmental goods are triggers for the emergence of cyclical evolutions in both the level of environmental quality and the economic variables. Because economic agents consider the evolution of the environment as given, they do not make any investment in environmental maintenance. Instead, they make self-protective choices through private consumption that can exacerbate environmental problems causing, in turn, feedbacks prone to the emergence of cyclical dynamics.^2^ This article aims at investigating whether environmental assets can be a cause of cyclical dynamics even in a fully decentralized setting where instead of considering the representative agent hypothesis, in which the environmental evolution is assumed as given (Antoci et al. 2007), there are interacting agents that can exhibit pro-environmental behaviors. In doing so, we extend the OLG model developed by John and Pecchenino (1994). In the original framework, the allocative problem is solved by short lived representative agents who act as social planners. The agents live two periods and choose whether saving their labor income to consume a private good when they are old or spending it for the environment. Unlike John and Pecchenino (1994), we consider that decisions are taken in a decentralized form by a population of agents each of which lives, without the possibility of migration, on a cell of a two-dimensional grid and makes decisions based on the level of environmental quality that is experienced on that cell. Consumption choices and expenditures to improve the environment in their own cells affect the environmental quality of nearby cells through externalities that cross the boundaries of the cell (a concept similar to the transboundary externalities among regions introduced in La Torre et al. (2021), but in our framework expressed as externalities among individuals). In the absence of interactions (no externalities among cells), the model collapses into a model formally similar to Zhang (1999). In this case, closed-form results concerning the existence and stability of stationary states can be obtained. As we mentioned above, even if cycles are proved to exist, when imposing values from the econometric literature to economic parameters, regardless of environmental parameters, the model exhibits dynamics that lead each cell to its long-term level or cycles with small periods (2-cycles or 4-cycles). Therefore, we investigate how the interaction between agents modifies the evolution of the system. This analysis connects our work also to the literature on the provision of public goods on networks. A large literature has studied in both static (see Bramoullé and Kranton 2007; Elliott and Golub 2019) and dynamic (Allouch 2015) contexts how the network structure and the links between agents may affect the characteristics of the Nash equilibrium related to the local public good game. Regarding the static context, starting from the pioneering work of Bergstrom et al. (1986), Bramoullé and Kranton (2007) develop a network model related to the provision of a public good which is non-excludable for agents connected in the same network. In terms of environmental contribution (and consequently of the levels of environmental good provided), they notice that as the size of the social network in which an individual is included increases, the probability of exerting a low effort for the environment increases. Thus, in the presence of very large networks, a high number of free riders are observed. Although we consider a network with a homogeneous structure, a similar phenomenon may be also observed in our model. In fact, (i) due to the presence of externalities caused by the choices of nearby agents and (ii) the overlapping generations structure of the model, the standard free riding problem arises both in intragenerational and intertemporal terms. Concerning the dynamic strand, Allouch (2015) analyzes the existence and stability of the Nash equilibrium in a network in which agents interact with respect to the consumption of a public good. The author shows that the higher the marginal propensities to consume the public good, the less consumers substitute and adjust to their neighbors’ provisions and then the Nash equilibrium is stable. In line with this result, in our work the presence of an overlapping generations structure (where agents optimize in a finite horizon setting while the environment evolves in infinite time) and interaction between agents induces a similar phenomenon. Considering the preference toward the local environment as the propensity to consume the public good, we notice that for high levels of preference the environmental dynamics will (statistically) tend to converge to Nash equilibrium. Further, the likelihood of converging to the stationary point increases in the case of heterogeneous agents. Our result is also in line with Corazzini and Gianazza (2008), who study a model where agents are distributed over a circle and contiguous agents interact locally, showing how high levels of preference for the environmental good are a vehicle for a stable Nash equilibrium. Considering the provision of public goods in a context of positive spillovers between jurisdictions, Bloch and Zenginobuz (2007) study a non-cooperative game between jurisdictions in which levels and asymmetries among spillovers play a key role in determining the value of the Nash equilibrium. With respect to this work, our model (i) allows us to observe that the equilibrium not only changes as the spillovers between interacting cells in the neighborhood vary, but for sufficiently high values of these spillovers the equilibrium loses stability; (ii) reinforces, in a dynamic context, the complexity result found by the authors regarding equilibrium levels for the provision of public goods when *n* jurisdictions (with $n>2$) are assumed. Indeed, the interaction among several agents (homogeneous or heterogeneous) in contiguous positions may give rise to continuous changes in allocative decisions.In terms of general results, the main findings of this article are that (i) the local dimension of the environmental good can justify the plausibility of agents participating in its maintenance; (ii) externalities between cells are the engine for the emergence of oscillatory dynamics. Indeed, comparison with Zhang (1999) shows that interactions between different generations cannot alone lead to nonlinear trends in environmental and economic variables if realistic parameter sets are considered; (iii) heterogeneity plays a stabilizing role in the dynamics because differentiated behavior among agents creates a more balanced grid in terms of environmental quality and economic outcomes, reducing feedback reactions by agents. However, this dampening effect is only partial; (iv) when externalities among cells exist, the environment’s inability to regenerate at high rates and the extent of the negative impact of consumption on the environment play a destabilizing role in the dynamics, while the effectiveness of environmental spending and a strong generalized interest or disinterest in the environment are elements that favor the stabilization of the dynamics.The remainder of the paper is organized as follows: Sect. 2 describes the structure of the OLG agent-based model; Sect. 3 provides an analysis of the properties of the model when local interactions are not allowed; Sect. 4 discusses the results of simulations performed when local interactions exist. Finally, Sect. 5 concludes.

## The model

We consider an overlapping generations economy where two generations, the *young* and the *old*, coexist at every discrete time period $t=1,\ldots ,+\infty$.^3^ The number of economic agents belonging to each generation is assumed to be constant and each agent lives, without the possibility of movement^4^, in a specific cell *v* of a lattice *L* with dimension $N\times \,N$. Therefore, there exists a one-to-one correspondence between the $N^{2}$ agents and the cells and we indicate the agent living in the cell *v* as the agent *v*. To avoid problems in defining neighborhoods at grid boundaries, we assume that *L* is wrapped to create a *torus* structure. By following John and Pecchenino (1994) and Naimzada and Sodini (2010), every agent *v* born at time *t* has preferences toward the consumption in the old age, $c_{t+1}^{v}$, and a positive index of the local environmental quality in the old age, $E_{t+1}^{v}$.^5^ Specifically, we assume the following logarithmic utility function

1 $$\begin{aligned} U^{v}(c_{t+1}^{v},E_{t+1}^{v})=\omega ^{v}\ln {c_{t+1}^{v}}+(1-\omega ^{v})\ln {E_{t+1}^{v}} \end{aligned}$$

where $\omega ^{v}\in (0,1)$ and $(1-\omega ^{v})$ represent the weights that individuals give, respectively, to consumption and environmental quality in their preferences. In what follows, to simplify the analysis, we introduce the elasticity parameter $\eta ^{v}=\frac{1-\omega ^{v}}{\omega ^{v}}$. During her youth, each agent *v* supplies inelastically her time endowment (which is normalized to 1) to the productive sector receiving a wage $w^{v}_{t}$ that she will divide between saving, $s_{t}$, for consumption when old, and investment in environmental maintenance, $m_{t}$, for improving the environmental quality of the cell *v* at $t+1$.

**Fig. 1:**
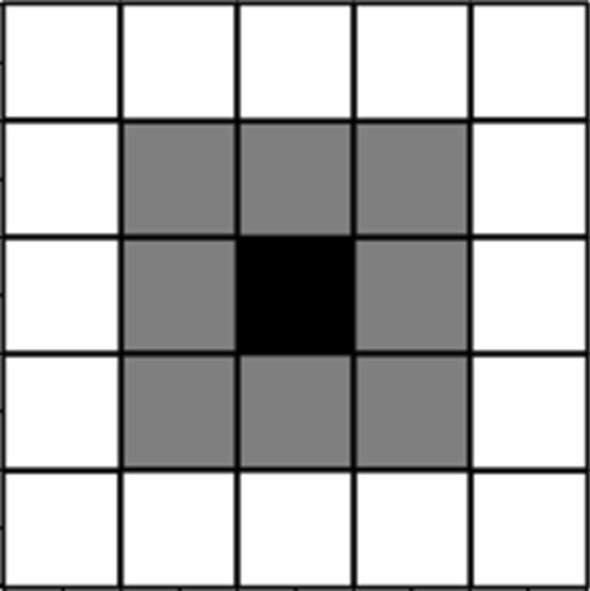
A sketch of the Moore neighborhood scheme. (i) The black cell in the grid represents the agent *v*, in the position (*i*, *j*) of *L*, with $i=1,..,N$ and $j=1,..,N$; (ii) the dark gray cells describe the neighborhood, that is agents positioned at (*n*, *p*) with $n=i-1,\ldots ,i+1$ and $p=j-1,\ldots ,j+1$ and $(n,p)\ne \,(i,j)$; (iii) the white cells define agents that do not belong in the immediate neighborhood of the agent in the black one

The consumption good is produced by *Z* perfectly competitive firms. Then, the output *Y* is produced according to the Cobb–Douglas technology

2 $$\begin{aligned} Y_{t}=Af(K_{t},N^{2})=A\,K_{t}^{\alpha }(N^{2})^{1-\alpha } \end{aligned}$$

where $K_{t}$ is the physical capital, *A* is a positive parameter representing the technological progress, and $\alpha \in (0,1)$ represents the elasticity of capital. By introducing the capital per worker $k_{t}=\frac{K_{t}}{N^{2}}$, the production function reads as

3 $$\begin{aligned} Y_{t}=y_{t}N^{2} \end{aligned}$$

where $y_{t}=Ak_{t}^{\alpha }$. Concerning the local environmental quality perceived by each agent, we assume that it depends not only on the decisions made by the agents of the various generations in that cell, but it is also affected by the actions of her contemporaries positioned on her *immediate* neighborhood. Specifically, we use a *Moore neighborhoods* scheme (see Shiflet and Shiflet 2014), in which any neighborhood is composed by nine cells: the central one indexed with *v* and the set of eight cells which surround it denoted with $I_{v}$ (see Fig. 1).

Adapting the specification employed in John and Pecchenino (1994) and Zhang (1999), we assume that the index of *local* environmental quality in the cell *v* evolves according to

4 $$\begin{aligned} E_{t+1}^{v}=(1-b)E_{t}^{v}+b\overline{E}^{v}+\bigg [\gamma ^{v}\,m_{t}^{v}+\sum _{l\in \,I_{v}} \gamma _{o}^{l}\,m_{t}^{l}\bigg ]-\bigg [\beta ^{v}\,c_{t}^{v}+\sum _{l\in \,I_{v}} \beta _{o}^{l}\,c_{t}^{l}\bigg ]. \end{aligned}$$

The parameter $\overline{E}^{v}>0$ represents the value toward the index tends when consumption and environmental expenditures are null, $b\in (0,1)$ measures the speed of reversion of the environmental quality to $\overline{E}^{v}$ and $E_{t}^{v}>0$ is the current level of the index in the cell *v*. The terms enclosed in the square brackets concern the impact of agents decisions on the future level of environmental quality experienced in the cell *v*: (i) in the first brackets, $\gamma ^{v}\,m_{t}^{v}$ and $\sum _{l\in \,I_{v}} \gamma _{o}^{l}\,m_{t}^{l}$ measure environmental improvement on the cell *v* induced by environmental expenditure of the young agent *v* and by the positive externalities produced by agents living in her neighborhood, respectively; (ii) in the second brackets, $\beta ^{v}\,c_{t}^{v}$ and $\sum _{l\in \,I_{v}} \beta _{o}^{l}\,c_{t}^{l}$ $\sum _{l\in \,I_{v}}$ measure the environmental damage caused on the cell *v* by the consumption choices of the agent *v* and her neighbors, respectively.^6^ Assuming that the choices made by agents *v* on the cell *v* generate higher impacts than those generated, on the cell *v*, by the choices made by the agents in $I_{v}$, we have $\gamma ^{v}>\gamma _{o}^{l}\ge \,0$ and $\beta ^{v}>\beta _{o}^{l}\ge \,0$ for every *v* and $l\in \,I_{v}$. In addition, we impose $\beta ^{v},\gamma ^{v}\le \,1$. This assumption, as shown by Zhang (1999), allows analyzing the dynamics of the model without loss of generality.

Agents supply their saving $s_{t}^{v}$ inelastically to firms and earn the gross return $(1+r_{t+1}-\delta )$ where $r_{t+1}$ is the real interest rate and $\delta$ is the depreciation rate of capital. Then, each individual *v* faces the following life-cycle budget constraints:

5 $$\begin{aligned}&c_{t+1}^{v}=(1+r_{t+1}-\delta )s_{t}^{v}; \end{aligned}$$

6 $$\begin{aligned}&\quad w_{t}^{v}=s_{t}^{v}+m_{t}^{v}; \end{aligned}$$

7 $$\begin{aligned}&\quad c_{t+1}^{v}>0, m_{t}^{v},s_{t}^{v}\ge \,0. \end{aligned}$$

Individual *v* maximizes the objective function

8 $$\begin{aligned} U^{v}(c_{t+1}^{v},E_{t+1}^{e,v}) \end{aligned}$$

under constraints (5)-(6)-(7), with respect to the choice variables $\,s_{t}^{v},\,m_{t}^{v},\,c_{t+1}^{v}$, taking $w_{t}$, $r_{t+1}$ as given. Assuming that the rule in (4) is known to the agents, the expected future environmental quality at $t+1$ for the agent *v* is given by

9 $$\begin{aligned} E_{t+1}^{e,v}=(1-b)E_{t}^{v}+b\overline{E}^{v}+\bigg [\gamma ^{v}\,m_{t}^{v}+\sum _{l\in \,I_{v}} \gamma _{o}^{l}\,m_{t}^{e,l}\bigg ]-\bigg [\beta ^{v}\,c_{t}^{v}+\sum _{l\in \,I_{v}} \beta _{o}^{l}\,c_{t}^{l}\bigg ].\ \ \ \ \ \ \end{aligned}$$

The term $\sum _{l\in \,I_{v}} \gamma _{o}^{l}\,m_{t}^{e,l}$ refers to the aggregate environmental improvement triggered by the expectations of the agent *v* on the defensive expenditures of her neighbors. In particular, we assume that every agent expects her neighbors to continue behaving as in the previous period (*naive expectations*), that is $m_{t}^{e,l}=m_{t-1}^{l}$ for every $l\in \,I_{v}$ and for every $t\ge \,1$.^7^ The last block in brackets collects consumption of the old agents in *v* and in $I_{v}$.^8^ As suggested by the condition in (7), we allow for the possibility that each agent *v* decides not to be involved in environmental maintenance ($m_{t}^{v}=0$). The interior solution of the problem, when it exists, is instead characterized by the first order condition

10 $$\begin{aligned} -U_{1}^{v}(c_{t+1}^{v},E_{t+1}^{e,v})(1+r_{t+1}-\delta )+\gamma ^{v}U_{2}^{v}(c_{t+1}^{v},E_{t+1}^{e,v})=0. \end{aligned}$$

From (7) and (10), we get the following individual optimal choices $(s_{t}^{v})^{*}$ and $(m_{t}^{v})^{*}$

11 $$\begin{aligned} (s_{t}^{v})^{*}&=\min \bigg (\frac{E_{t+1}^{e,v}}{\gamma ^{v}\eta ^{v}},w_{t}^{v}\bigg )\nonumber \\&= \min \bigg (\frac{(1-b)E_{t}^{v}+b\overline{E}^{v}+\gamma ^{v}\,w_{t}^{v}+\Lambda -\beta ^{v}\, (c_{t}^{v})^{*}-\Psi }{\gamma ^{v}(\eta ^{v}+1)},w_{t}\bigg ); \end{aligned}$$

12 $$\begin{aligned}&\quad (m_{t}^{v})^{*}=\max \bigg (0,w_{t}^{v}-(s_{t}^{v})^{*}\bigg ); \end{aligned}$$

where $$(c_{t}^{v})^{*}=(1+r_{t}-\delta )(s_{t-1}^{v})^{*}$$, $$\Lambda =\sum _{l\in \,I_{v}} \gamma _{o}^{l}\,(m_{t-1}^{l})^{*}$$ and $$\Psi =\sum _{l\in \,I_{v}} \beta _{o}^{l}\,(c_{t}^{l})^{*}$$.^9^

The goods market clearing condition reads as

13 $$\begin{aligned} N^{2}\,k_{t+1}=\sum _{v=1}^{N^{2}} s_{t}^{v}. \end{aligned}$$

At each period *t*, firms maximize their profit, and then the following equilibrium equations for wage and interest rate are obtained:

14 $$\begin{aligned}&w_{t}^{v}=w_{t}=(1-\alpha )Ak_{t}^{\alpha }; \end{aligned}$$

15 $$\begin{aligned}&\quad r_{t}=\alpha \,A\,k_{t}^{\alpha -1}. \end{aligned}$$

From the solutions of the maximization problem for the agent *v* in (11)-(12), the equilibrium expressions for wage and real interest rate in (14)-(15) and equations (4)-(13), then the equilibrium dynamics for every *v* are defined by the following system of first order difference equations:

16 $$\begin{aligned} J: {\left\{ \begin{array}{ll} k_{t+1}=\min \bigg (\frac{(1-b)E_{t}^{v}+b\overline{E}^{v}+\gamma ^{v}\,A(1-\alpha )k_{t}^{\alpha }+\Lambda -\beta ^{v}\,c_{t}^{v}-\Psi }{\gamma ^{v}(\eta ^{v}+1)},A(1-\alpha )k_{t}^{\alpha }\bigg )\\ E_{t+1}^{v}=(1-b)E_{t}^{v}+b\overline{E}^{v}+\bigg [\gamma ^{v}\,(m_{t}^{v})^{*}+\Lambda ^{*}\bigg ]-\bigg [\beta ^{v}\,(c_{t}^{v})^{*}+\Psi \bigg ] \end{array}\right. }\nonumber \\ \end{aligned}$$

where $$\Lambda ^{*}=\sum _{l\in \,I_{v}} \gamma _{o}^{l}\,(m_{t}^{l})^{*}$$.

### Bounds to $E^{v}$ dynamics

We notice that if agents do not contribute to the environmental defense, the evolution of state variables in the generic cell *v* is described by the following equations:

17 $$\begin{aligned} {\left\{ \begin{array}{ll} E_{t+1}^{v}=(1-b)E_{t}^{v}+b\overline{E}^{v}-\beta ^{v} (c_{t}^{v})^{*}-\sum _{l\in \,I_{v}} \beta _{o}^{l}\,c_{t}^{l}\\ k_{t+1}=w_{t}=A(1-\alpha )k_{t}^{\alpha }. \end{array}\right. }\nonumber \\ \end{aligned}$$

The dynamics of *k* autonomously evolve and converge to the stationary equilibrium

18 $$\begin{aligned} k_{\max }^{*}=(A(1-\alpha ))^{\frac{1}{1-\alpha }}. \end{aligned}$$

Because the expression $k_{t+1}=w_{t}-m_{t}\le w_{t}$ holds, $k_{\max }^{*}$ also defines the highest value that *k* may assume given an initial condition $k_{0}<k_{\max }^{*}$, for any initial value $E^{v}_{0}$ and any sequence of decisions made by agents. Thus, in the generic cell *v*, $E^{v}$ dynamics satisfy the following inequality

19 $$\begin{aligned} E_{t+1}^{v}\ge \,(1-b)E_{t}^{v}+b\overline{E}^{v}-(\beta ^{v}+8\beta _{O})c_{\max } \end{aligned}$$

where $\beta _{O}=\max (\beta _{o}^{l})$ with $l\in \,I_{v}$ and $c_{\max \text { }}=\frac{(A(1-\alpha ))^{\frac{1}{1-\alpha }}(1-\delta (1-\alpha ))}{1-\alpha }$ is the maximum value of consumption for $k_{t}\in \left[ 0,(A(1-\alpha ))^{\frac{1}{1-\alpha }}\right]$. In this case, the dynamics described by $E_{t+1}^{v}=(\text {\textit{RHS} of}$ (19)) converge to

20 $$\begin{aligned} (E_{\min }^{v})^{*}:=\overline{E}^{v}-\frac{(A(1-\alpha ))^{\frac{1}{1-\alpha }} (1-\delta (1-\alpha ))(\beta ^{v}+8\beta _{O})}{(1-\alpha )b}<\overline{E}^{v} \end{aligned}$$

which represents the lowest value that $E^{v}$ may exhibit. In what follows, we assume that the initial condition $E_{0}^{v}$ is not too far from $\overline{E}^{v}$, that is we assume that at the beginning of the anthropic activity the environment is close to its stationary value.

To avoid cases in which the index $E^{v}$ takes negative values, that would not be consistent with the definition of the utility function, we consider the expression in (20) and we assume that

21 $$\begin{aligned} \overline{E}^{v}\ge \,\overline{E}_{\min }^{v}=\frac{1-\delta (1-\alpha )}{b}A^{\frac{1}{1-\alpha }}(1-\alpha )^{\frac{\alpha }{1-\alpha }}(\beta ^{v}+8\beta _{O}). \end{aligned}$$

Let us now consider the case when agents decide to devote their entire wage to the environmental defense. The evolution of state variable $E^{v}$ in the generic cell *v* becomes

22 $$\begin{aligned} E_{t+1}^{v}=(1-b)E_{t}^{v}+b\overline{E}^{v}+\gamma ^{v} m_{t}^{v}+\sum _{l\in \,I_{v}} \gamma _{o}^{l}\,m_{t}^{l} \end{aligned}$$

and the following inequality holds:

23 $$\begin{aligned} E_{t+1}^{v}\le \,(1-b)E_{t}^{v}+b\overline{E}^{v}+(\gamma ^{v}+8\gamma _{O})m_{\max } \end{aligned}$$

where $\gamma _{O}=\max (\gamma _{o}^{l})$ with $l\in \,I_{v}$ and $m_{\max }=A(1-\alpha )\big [(A(1-\alpha ))\big ]^{\frac{1}{1-\alpha }}$.

In this case, a higher private wealth allows agents to invest a higher amount of resources in the environmental maintenance. From the equation of capital accumulation and (23), it follows that

24 $$\begin{aligned} E_{t}^{v}\le \,(E_{\max }^{v})^{*}:=\overline{E}^{v}+\frac{(\gamma ^{v}+8\gamma _{O})A(1-\alpha )\big [(A(1-\alpha ))\big ]^{\frac{1}{1-\alpha }}}{b},\ \ \ \ \forall \,t. \end{aligned}$$

The level $(E_{\max }^{v})^{*}$ defined in (24) will be used in the simulation analyses performed in the following sections to define a normalized index of environmental quality $\widetilde{E}_{t}^{v}\in (0,1)$ with

25 $$\begin{aligned} \widetilde{E}^{v}_{t}=\frac{E^{v}_{t}}{(E_{\max }^{v})^{*}}. \end{aligned}$$

In this regard, we notice that the agent’s allocative choices are unaffected if we consider the following expression of the utility function

26 $$\begin{aligned} U^{v}(c^{v}_{t+1},\widetilde{E}^{v}_{t+1})=\omega ^{v}\ln {c_{t+1}^{v}}+(1-\omega ^{v})\ln {\widetilde{E}_{t+1}^{v}}. \end{aligned}$$

The usefulness of using this normalized index lies in the fact that the range over which $E^{v}_{t}$ varies is strongly conditioned by the model parameters especially by *b*, making it difficult to compare dynamics generated by different parameter values.^10^

## The model without local interactions

If we assume no local interactions among individuals (that is, $\beta _{o}^{l}=\gamma _{o}^{l}=0$ for every $l\in \,I_{v}$), the dynamic system in a generic cell *v* reads as

27 $$\begin{aligned} M: {\left\{ \begin{array}{ll} E_{t+1}=(1-b)E_{t}+b\overline{E}-\beta \,c_{t}+\gamma \,m_{t}\\ k_{t+1}=\min \,\bigg (\frac{E_{t+1}}{\gamma \,\eta },w_{t}\bigg ) \end{array}\right. } \end{aligned}$$

where *M* describes the dynamics on every generic cell *v* of the lattice *L*, independently by the allocative choices made in its Moore neighborhood. Moreover, we can notice that, with this assumption, in every cell of *L* the expected and realized future environmental levels are equal ($E_{t+1}^{e}=E_{t+1}$). It means that, when an interior solution exists, capital and environmental dynamics are proportional at each time period following the equation in (11). Then, the analysis of the map *M* collapses in studying the unidimensional map

28 $$\begin{aligned} H:E_{t+1}:=f(E_{t})=(1-b)E_{t}+b\overline{E}-\beta \,c_{t}+\gamma \,m_{t}^{*}. \end{aligned}$$

For the study of the map (28), it is convenient to start from the case $\overline{E}=0$. With this assumption, the map *H* may be rewritten as

29 $$\begin{aligned} \widetilde{H}:\widetilde{f}(E_{t})=a_{0}E_{t}+a_{1}(E_{t})^{\alpha } \end{aligned}$$

where $a_{0}=\frac{(1-b)\eta \gamma -\beta (1-\delta )}{\gamma (1+\eta )}$ and $a_{1}=\frac{A(\eta )^{1-\alpha }[\gamma (1-\alpha )-\beta \alpha ]}{(\gamma )^{\alpha }(1+\eta )}$. By considering the map (29), we get the following results:

### Proposition 1

Let $\widetilde{H}$ be the map defined in (29). If $\gamma \le \frac{\beta \alpha }{1-\alpha }$, then the map admits the unique fixed point $E^{*}_{1}=0$. Otherwise, the map admits both the fixed point $E^{*}_{1}=0$ and a positive fixed point $E^{*}_{2}$.

### Proof

The result follows by solving the equation $f(E_{t})=E_{t}$. The positive solution $E^{*}_{2}$ is given by

30 $$\begin{aligned} E^{*}_{2}=\bigg (\frac{a_{1}}{1-a_{0}}\bigg )^{\frac{1}{1-\alpha }} \end{aligned}$$

which exists if and only if $\frac{a_{1}}{1-a_{0}}>0$. Being immediate to verify that $a_{0}<1$ in the whole parameter space, the result is obtained by the condition which guarantees that $a_{1}>0$. $\square$

### Proposition 2

Let $\widetilde{f}$ be the function defined in (29). If $\gamma >\frac{\beta \alpha }{1-\alpha }$, then $\widetilde{f}$ is strictly concave and admits the fixed point $E^{*}_{2}$.

### Proof

By computing the second derivative

31 $$\begin{aligned} \widetilde{f}^{\prime \prime }(E_{t})=\alpha (\alpha -1)a_{1}(E_{t})^{\alpha -2}, \end{aligned}$$

we find that $\widetilde{f}^{\prime \prime }(E_{t})<0$ if and only if $a_{1}>0$. Then, the result follows.

$$\square $$

Concerning the stability of the fixed points $E^{*}_{1}$ and $E^{*}_{2}$, the following results are stated:

### Proposition 3

Let $\widetilde{f}$ be the function defined in (29).

(i) The fixed point $E_{1}^{*}$ is always unstable;
(ii) if $\eta >\frac{(1-\delta )\beta (1-\alpha )-\gamma (1+\alpha )}{\gamma (2+b(\alpha -1))}$, then $E_{2}^{*}$ is locally asymptotically stable. Otherwise, $E_{2}^{*}$ is unstable.

### Proof

The results follow by evaluating the first derivative

32 $$\begin{aligned} f^{\prime }(E_{t})=a_{0}+a_{1}\alpha \,(E_{t})^{\alpha -1} \end{aligned}$$

at the stationary states. Being $\alpha \in (0,1)$, it is straightforward that $\lim \limits _{E\rightarrow \,0^{+}} \widetilde{f}^{\prime }(E)>1$. The result in (ii) follows by solving the inequality

33 $$\begin{aligned} -1<a_{0}+a_{1}\alpha \,(E^{*}_{2})^{\alpha -1}<1. \end{aligned}$$

$$\square $$

If we assume $\overline{E}>0$, and $\gamma >\frac{\alpha \beta }{1-\alpha }$, the map in (28) preserves the concavity property described in Proposition 2. Then, we obtain the following result:

### Proposition 4

Let assume $\gamma >\frac{\alpha \beta }{1-\alpha }$ and $\overline{E}>0$. The map *H* admits a unique fixed point $E^{*}$.

### Proof

We can notice that $f(0)=b\overline{E}>0$. This implies that the graph of the map *H* crosses the 45-degree line at least once. Due to the concavity of *H* ensured by the condition $\gamma >\frac{\alpha \beta }{1-\alpha }$, the intersection point is unique.

$$\square $$

Although the expression of the fixed point for *H* cannot be found analytically, the following Proposition classifies its stability:

### Proposition 5

Let assume $\gamma >\frac{\alpha \beta }{1-\alpha }$ and *H* be the map defined in (28). (a) If $\frac{(1-\delta )\beta (1-\alpha )-\gamma (1+\alpha )}{\gamma (2+b(\alpha -1))}<\eta <\frac{\beta (1-\delta )-\gamma }{\gamma \,(2-b)}$, then there exists a threshold value $\overline{E}_{th}$ such that for $\overline{E}<\overline{E}_{th}$ $E^{*}$ is locally asymptotically stable, while for $\overline{E}>\overline{E}_{th}$, $E^{*}$ is unstable. (b) If $\eta <\frac{(1-\delta )\beta (1-\alpha )-\gamma (1+\alpha )}{\gamma (2+b(\alpha -1))}$, then $E^{*}$ is unstable for every $\overline{E}>0$.

### Proof

It is straightforward to prove that, under the assumption $\gamma >\frac{\alpha \beta }{1-\alpha }$, there is a positive monotonic relationship between $\overline{E}$ and $E^{*}$ and, as $\overline{E}$ tends to $+\infty$, $E^{*}$ tends to $+\infty$. Considering the first derivative $f^{\prime }(E^{*})=a_{0}+a_{1}\alpha \,(E^{*})^{\alpha -1}$, we notice that $f^{\prime }$ is monotonically decreasing. (a) For the assumption on $\eta$, we get $f^{\prime }(E^{*})|_{\overline{E}=0}\in (-1,1)$ and $\lim \limits _{\overline{E}\rightarrow \,+\infty }f^{\prime }(E^{*})=a_{0}<-1$. This implies that there exists a threshold value $\overline{E}_{th}$ such that $f^{\prime }(E^{*})=-1$. Hence, the result follows. (b) In this case, for $\overline{E}=0$, $f^{\prime }(E^{*})<-1$ and due to the monotonic behavior of $f^{\prime }(E^{*})$, the result trivially follows. $\square$

Concerning the role of the value $\overline{E}_{th}$, the following Corollary holds:

### Corollary 6

At the value $\overline{E}=\overline{E}_{th}$, the map undergoes a Flip bifurcation.

**Fig. 2:**
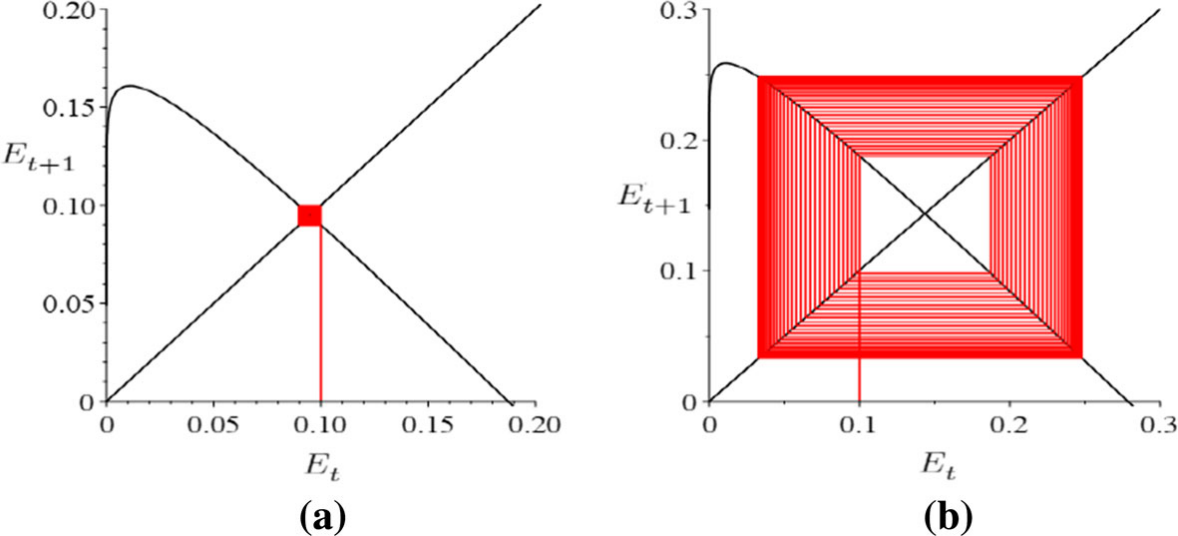
Stability **a** and instability **b** of the fixed point $E^{*}$ for $\overline{E}<\overline{E}_{th}$ and $\overline{E}>\overline{E}_{th}$, respectively

The stability properties stated in the previous Proposition can be highlighted by the following numerical exercise. Specifically, Fig. 2 shows that, considering the parametric set $\alpha =0.1,\gamma =0.11,\delta =0.016,\eta =0.8,A=5,b=0.22,\beta =0.3$, the map *H* admits a single positive fixed point. Drawing the first 100 iterations of the map (depicted in red), Panel (a) shows that, for $\overline{E}=0.5$, the fixed point becomes stable, while Panel (b) displays that the fixed point is unstable, for a sufficiently high value of $\overline{E}$ ($\simeq \,1.5$). If the concavity property of $\widetilde{f}$ fails, the following result trivially holds:

### Proposition 7

If $\gamma <\frac{\alpha \beta }{1-\alpha }$, then the function $\widetilde{f}$ is always decreasing and the fixed point $E^{*}$ is always stable or the dynamics are captured by a 2-cycle for a sufficietly high value of $\overline{E}$.

We notice that, regardless of other parameters, $\overline{E}$ has a destabilizing role in the dynamics. This is due to the fact that a high level of $\overline{E}$ induces agents not to invest in the environment. However, because of the non-coordination across generations in the same cell, this can create such a low level of environmental quality for subsequent generations that this new level induces them to make a strong change in decisions on wage allocation. This situation is prone to create oscillatory dynamics.

Nevertheless, we anticipate (see the following Section) that for realistic values of the economic parameters the dynamics tend to converge to stationary equilibrium or a cycle of period 2. In the following section, we explore the properties of the model when the hypothesis of no externalities among cells is removed.

## The model with local interactions

In this section, we investigate the dynamics of the model when interactions among cells are considered. To reduce the complexity of the model analysis due to the presence of many parameters, we set the parameters related to the production sector and capital accumulation to values in line with those estimated in the econometric literature (that is, $A=9000$, $\alpha =0.33$ and $\delta =0.7870898627$^11^) and focus on the analysis of the role of environmental parameters in defining the evolution of the model. In any case, varying the parameter *A* in a range that gives rise to capital accumulation levels typical of advanced countries^12^, and/or varying the annual rate of technological obsolescence in a range between 0.05 and 0.08 (see Escribá-Pérez et al. 2018), and/or considering productivity in the range (0.25, 0.35) the results remain very close to those illustrated below. Moreover, considering that agents in every cell have access to the same production technology and the same consumption good, it is natural to assume that also the environmental damage parameter $\beta ^{v}$ and the defensive expenditure efficiency parameter $\gamma ^{v}$ remain constant in all the cells of the lattice ($\beta ^{v}=\beta$, $\gamma ^{v}=\gamma$), as well as the externalities they generate on neighboring cells ($\beta _{o}^{l}=\beta _{o}$,$\gamma _{o}^{l}=\gamma _{o}$). Finally, we assume that the environmental quality has the same characteristics over the whole grid. Then, we impose that $\overline{E}^{v}$ assumes the same value in all the cells (that is, $\overline{E}^{v}=\overline{E}$). As illustrated in Sect. 3, a lower bound for $\overline{E}$ has to be imposed in order to obtain well-defined dynamics. In particular, we set $\overline{E}=\overline{E}_{\min }^{v}$, where $\overline{E}_{\min }^{v}$ is defined in (20). By applying this condition on $\overline{E}$, the index of environmental quality steadily decreases to 0 if agents do not invest in environmental expenditures and use all the wage for consumption. In order to have an index that lies in the range (0, 1) for all the parameter setting, in what follows, we will refer to $\widetilde{E}^{v}_{t}$, defined in (25).^13^ Clearly, by imposing this scalarization, two different economies converging to the same value may be associated with two very far environmental results. Differences in $E^{v}_{t}$ may be due, at least in part, to exogenous factors randomly determined: the ability of nature to regenerate, *b*, and to the extent of the impacts of the agents’ allocations on the environment (i.e., the parameters $\beta$, $\beta _{o}$, $\gamma$, $\gamma _{o}$). In other words, environmental parameters define alternative and randomly selected states of the world in which agents make their decisions. Contrasting environmental dynamics associated with different states of the world can be really misleading. Instead, by considering the variable $\widetilde{E}^{v}_{t}$, trajectories of $\widetilde{E}^{v}_{t}$ close to 0 clearly describe a case of agents devoting few resources to the environment. Dynamics of $\widetilde{E}^{v}_{t}$ close to 1 identify cases where agents have invested greatly in the environment, regardless of the specific state of the world.

The Table 1 summarizes the assumptions on the parameters.

**Table 1:** Parameters of the model

Parameter	Baseline value or range	Source
*A*	9000	Capital stock data (FRED)
*b*	(0, 1)	See Eq. (4)
$\alpha$	0.33	Krueger (1999) and Gollin (2002)
$\beta ,\beta _{o}$	[0, 1] with $\beta _{o}<\beta$	See the description of the model
$\gamma ,\gamma _{o}$	[0, 1] with $\gamma _{o}<\gamma$	See the description of the model
$\delta$	0.7870898627	Escribá-Pérez et al. (2018)
$\overline{E}^{v}$	$\overline{E}_{\min }^{v}$	See main text
$\omega ^{v}$	(0, 1)	See Eq. (1)

Analytical classification of the dynamics generated by map *J* appears rather complicated. Therefore, to identify the dynamical properties of the model we will base our analysis on numerical simulations. Specifically, we will address the analysis of the model through Monte Carlo simulations with a sufficiently large number of iterations (2000).^14^ For each replication, by randomly drawing a set of parameters within the ranges described in Table 1, we let the dynamic run for 300 periods, starting from an initial condition $E_{0}$ close to the $\overline{E}$ value (i.e., the long-run value of *E* without anthropogenic activity) and for an initial capital level positive but lower than $k_{\max }$ ($k_{0}=0.0001$ in all the simulations).^15^ In what follows, we analyze the presence of convergent dynamics or of long-term cyclical dynamics, that is, dynamics producing oscillations after a transient of 100 periods. In addition, to characterize the type of oscillations that are produced in the various Monte Carlo runs, we consider the distribution of the variable $\Delta ^{\widetilde{E}^{v}}=\max {\widetilde{E}^{v}_{r}}-\min {\widetilde{E}^{v}_{s}}$ with $r,s>100$ that is the range over which the variable $E_{t}^{v}$ varies after the transient, regardless of the value around which this variation is generated (high values or low values of $\widetilde{E}^{v}$). Moreover, since the oscillatory dynamics are persistent, both $\max {\widetilde{E}^{v}_{r}}$ and $\min {\widetilde{E}^{v}_{s}}$, or values close to them tend to repeat more and more times. So, a higher value of the variable $\Delta ^{\widetilde{E}^{v}}$ can be interpreted as associated to a heavier oscillatory phenomenon. Alternative measures for the variability have been considered (as the coefficient of variation) obtaining results in line with those outlined in the remainder of the article.^16^ The role of grid size varies depending on the assumptions we are going to explore. In particular, *N* is irrelevant if agents are homogeneous. Each agent is surrounded by agents that replicate her choices for each value of *N*. In the case where agents are heterogeneous, a specific analysis on the role of *N* will be carried out (see Sect. 4.3).

### Local interactions make the difference

**Fig. 3:**
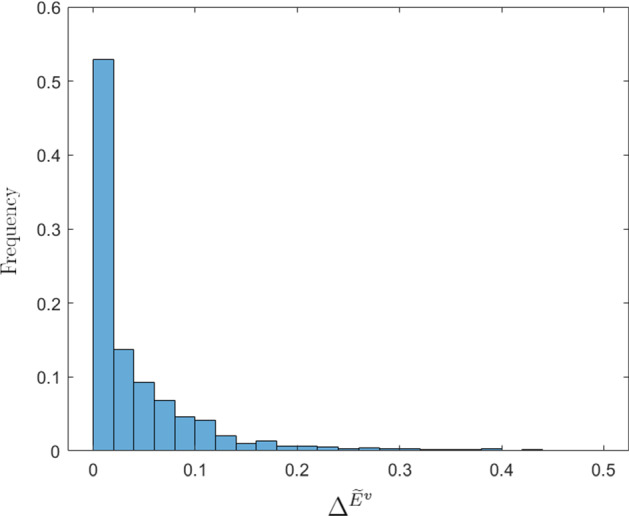
Distribution of $\Delta ^{\widetilde{E}^{v}}$ in the 2000 simulations

We begin our analysis by investigating the role of local interaction among individuals in the case of homogeneous agents. In Sect. 3, we highlighted that even in the case without interactions, oscillatory dynamics are possible. However, these results are possible only in the presence of parametric sets with values of economic parameters far from those estimated in the economic literature. As shown in Table 2, in 2000 replications of the model with $N=10$, without interactions among cells,^17^ only in $1\%$ of cases there are oscillations. The result dramatically changes with positive values of $\beta _{o}$ and $\gamma _{o}$. In this last case, the oscillations occur in $78\%$ of cases and, as shown in the histogram in Fig. 3, in about $48\%$ of cases the oscillations of the (normalized) environmental quality index have an amplitude greater than 0.1.

**Table 2:** The role of local interactions

Parameters	Convergent dynamics	$\%$ Cycles
$\beta _{o},\gamma _{o}=0$	1981	$1\%$
$0<\beta _{o}<\beta$, $0<\gamma _{o}<\gamma$	438	$78\%$

The economic interpretation of this result lies in the fact that even if we consider a local environmental variable and each agent is interested only in what happens in her cell, externalities create bridges between cells and the non-coordination between agents produces non-optimal allocations. Therefore, agents of subsequent generations may be pushed to change their allocative choices. If, for example, agent *v* finds a high environmental quality, she will tend to invest little in environmental expenditure at her cell. The worsening of the environment is, however, greater than expected, because the neighbors will also replicate her behavior. At that point, the new young agent in cell *v* will tend to increase the level of environmental expenditures, as well as her neighbors. This phenomenon is prone to create cyclical dynamics. The result is exacerbated by the fact that all agents are homogenous and live in cells characterized by the same level of environmental quality, and therefore, their allocations move in the same direction.

**Fig. 4:**
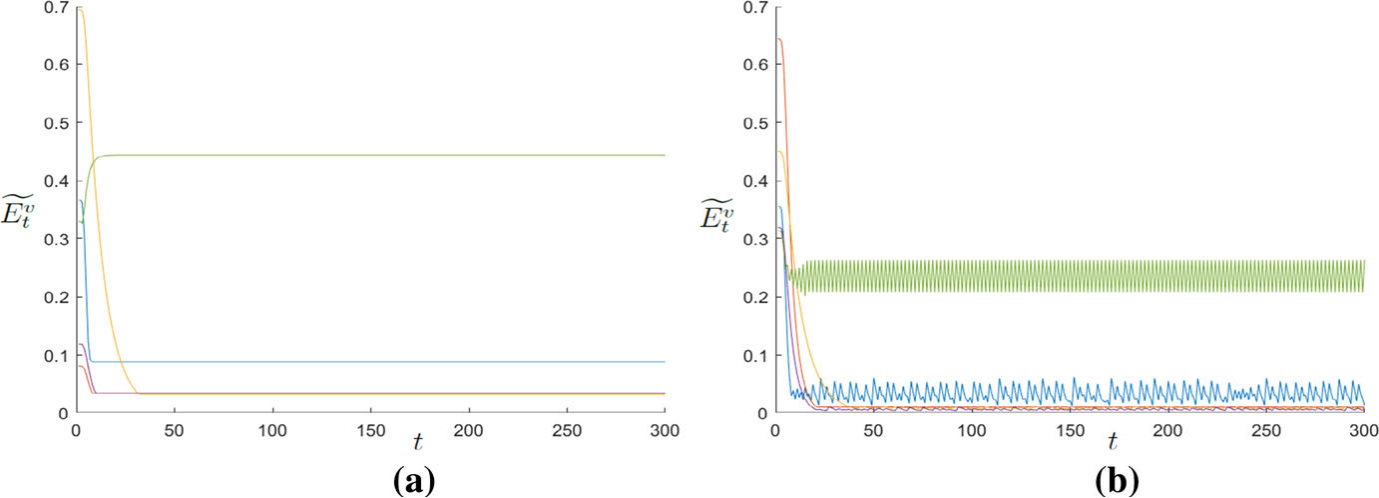
**a** Time series of $\widetilde{E}^{v}$ with no local interactions. **b** Time series of $\widetilde{E}^{v}$ with local interactions. For reasons of readability of the graph, only five time series have been shown

In order to exemplify what described, Fig. 4 shows in the Panels (a) and (b) four simulations without and with interaction, respectively, for the same parametric set. While Panel (a) shows that the dynamics of $\widetilde{E}^{v}$ without interaction converge to values dependent on the level of $\omega$ (the possibility of cycles is very rare), Panel (b) shows the onset of dynamics largely cyclical caused by the interactions among agents.

### Sensitivity analysis on environmental parameters

In this subsection, we study the role of environmental parameters in defining the dynamics of the model, to understand which parameters favor convergent dynamics and which favor oscillatory ones. Specifically, for every environmental parameter (that is, $\omega ,\beta ,\gamma ,\beta _{o},\gamma _{o},b$) we will perform a sensitivity analysis exercise. In particular, we will consider 9 increasing values of the parameter under analysis, from its lowest to its highest value as specified in Table 1 and for each of these values, we will perform a Monte Carlo analysis with the characteristics specified at the beginning of the section.

**Fig. 5:**
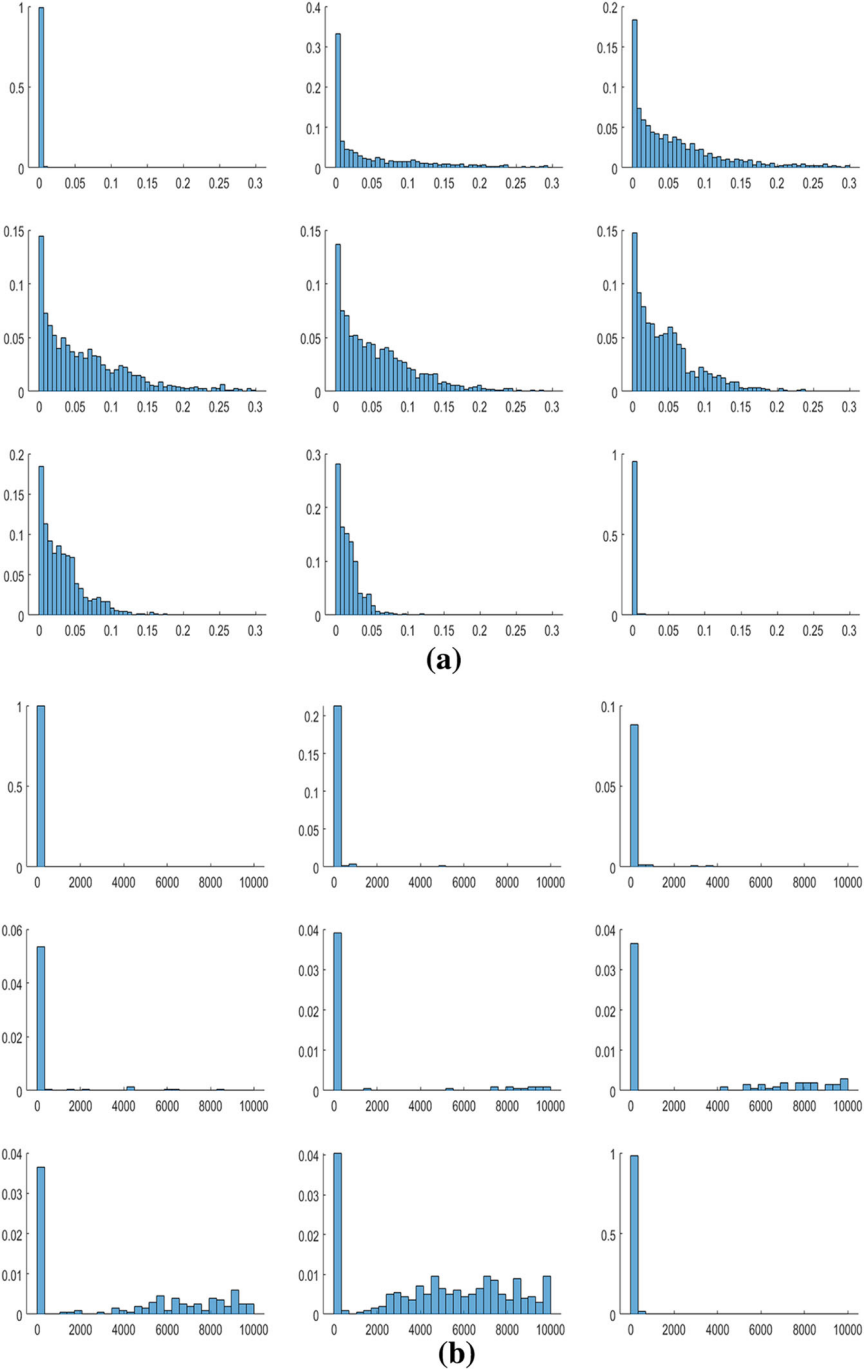
**a** Distribution of $\Delta ^{\widetilde{E}^{v}}$ as $\omega$ increases. **b** Distribution of $\Delta ^{k}$ as $\omega$ increases

**Fig. 6:**
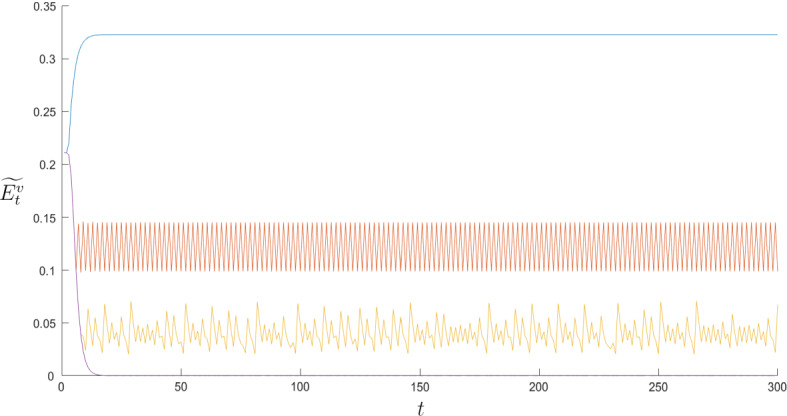
Time series of $\widetilde{E}^{v}$ as $\omega$ varies. Parameters: $\omega =0.001$ (curve in blue), $\omega =0.3337$ (curve in orange), $\omega =0.6663$ (curve in yellow), $\omega =0.999$ (curve in violet) (colour figure online)

Regarding the parameter $\omega$, for very low values of the parameter, the environmental dynamics, as well as capital accumulation, tend to converge to a long-run value. As the value of the parameter increases, the variability of the simulations increases, making it possible to observe a large number of oscillatory dynamics, and then, as the value further increases, the variability of the dynamics decreases, until a return to convergent dynamics is observed for very high values of the parameter (see Fig. 5). The reason for this result lies in the fact that $\omega$ describes the agents’ interest in the environment. If the level of $\omega$ is very high or very low, the externalities caused by the behaviors of neighboring agents are somewhat irrelevant. To understand this point, we can refer to the boundary cases $\omega =0$ and $\omega =1$: in the first case, regardless of the behavior of neighbors, agents are not interested in consumption and invest all resources in the environment. Then, $E^{v}$ tends to $(E_{\max }^{v})^{*}$ and *k* tends to 0. Instead, in the second case, the environmental quality is not evaluated by the agents at all and they will invest all their income in consumption. $E^{v}$ tends to 0 and *k* tends to $k_{\max }^{*}$. Therefore, for values of $\omega$ close to 0 and 1 the dynamics of $\widetilde{E}^{v}$ will stabilize. The evolutions of agents’ decisions become more complex in the case of intermediate values of $\omega$, where the behavior of neighbors can cause overall results that subsequent generations want to modify (an example of the dynamics observed as $\omega$ increases is shown in Fig. 6).

**Fig. 7:**
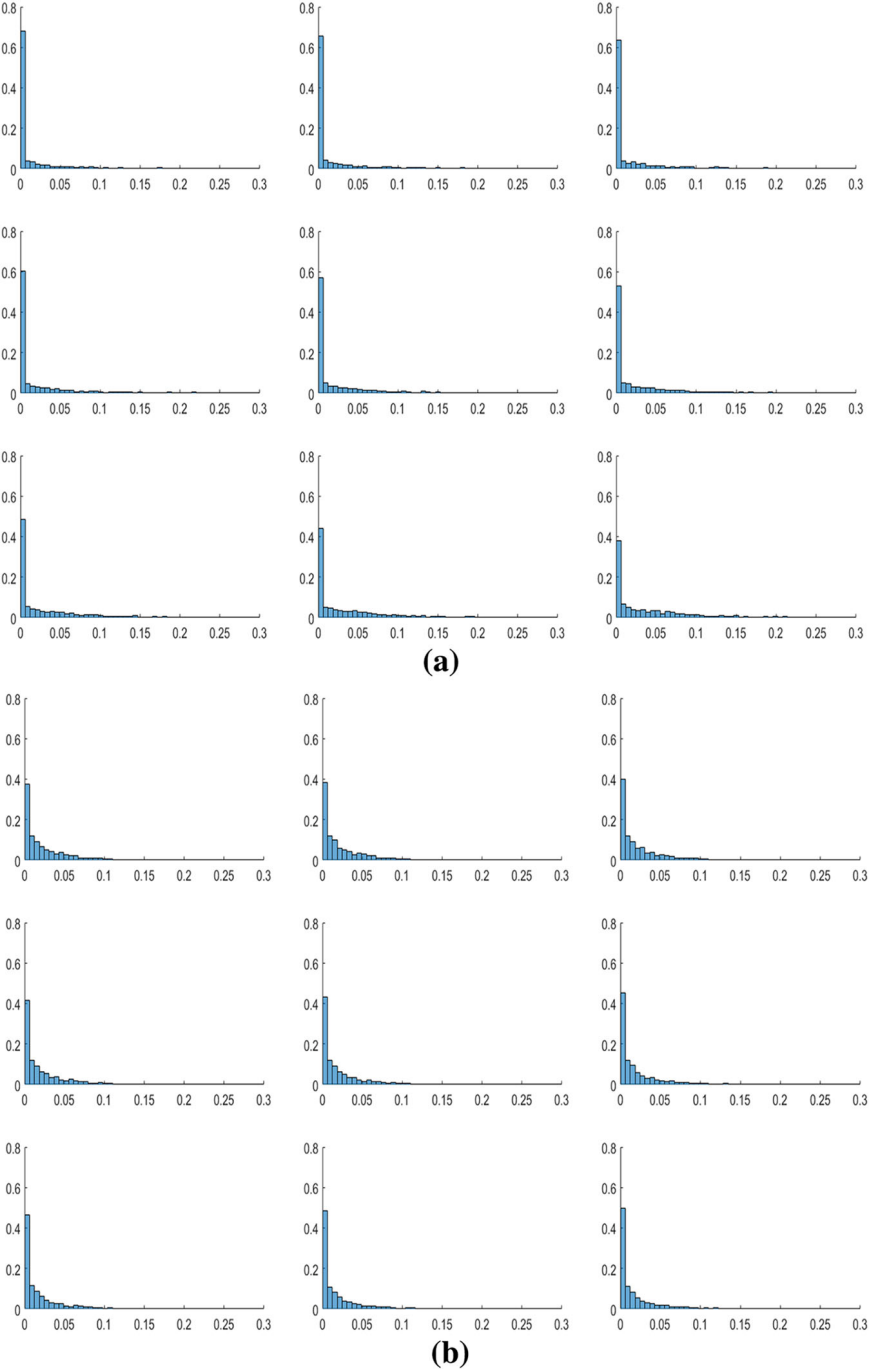
**a** Distribution of $\Delta ^{\widetilde{E}^{v}}$ as $\beta$ increases. **b** Distribution of $\Delta ^{\widetilde{E}^{v}}$ as $\gamma$ increases

**Fig. 8:**
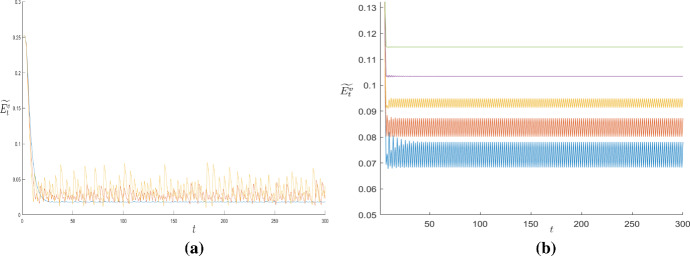
**a** Time series of $\widetilde{E}^{v}$ as $\beta$ increases. Parameters: $\beta =0.5$ (curve in blue), $\beta =0.7495$ (curve in orange), $\beta =0.999$ (curve in yellow). **b** Time series of $\widetilde{E}^{v}$ as $\gamma$ increases. Parameters: $\gamma =0.5$ (curve in blue), $\gamma =0.6248$ (curve in orange), $\gamma =0.7495$ (curve in yellow), $\gamma =0.8743$ (curve in violet), $\gamma =0.999$ (curve in green) (colour figure online)

The sensitivity analysis on the environmental parameters of the model also allows us to outline the opposite role that the two technological parameters $\beta$ and $\gamma$ have on the dynamics. In fact, as we show in the histograms in Fig. 7 and time series depicted in Fig. 8, if for increasing values of $\beta$ the variability in the simulations tends to increase, the reverse happens when the parameter $\gamma$ varies. These results robustly confirm what has been described in Zhang (1999). If consumption activity produces a strong environmental impact, subsequent generations will change their wage allocation decisions, investing more in environmental quality. This triggers a reduction in the savings rate and thus a reduction in economic resources for agents. This sequence of feedbacks is prone to generate oscillatory dynamics. Conversely, a high value of $\gamma$ generates a self-reinforcing system, which favors the stabilization of dynamics. The reason is that in this case, economic growth is positively and strongly correlated with environmental quality and the growth process does not generate a negative feedback.

**Fig. 9:**
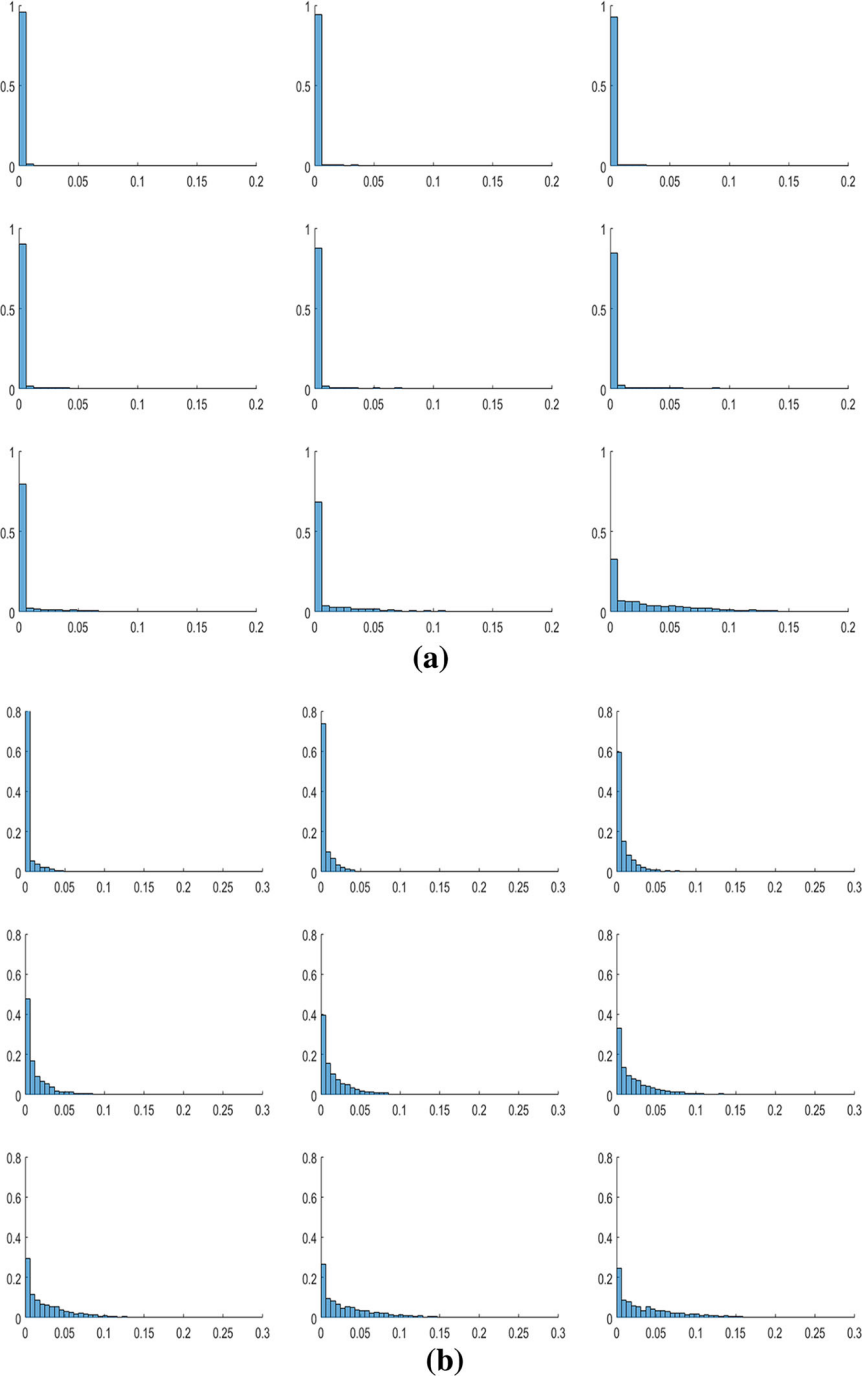
**a** Distribution of $\Delta ^{\widetilde{E}^{v}}$ as $\beta _{o}$ increases. **b** Distribution of $\Delta ^{\widetilde{E}^{v}}$ as $\gamma _{o}$ increases

Concerning the parameters of indirect impact on the local environment, i.e., $\beta _{o}$ and $\gamma _{o}$, we instead notice a univocal effect on the simulations. In fact, as both $\beta _{o}$ and $\gamma _{o}$ increase, the variability of the dynamics of $\widetilde{E}^{v}$ (and of the dynamics of *k* associated with it) increases (see Fig. 9).

**Fig. 10:**
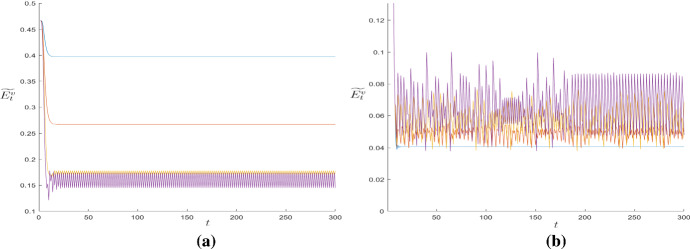
**a** Time series of $\widetilde{E}^{v}$ as $\beta _{o}$ increases. Parameters: $\beta _{o}=0.001$ (curve in blue), $\beta _{o}=0.167$ (curve in orange), $\beta _{o}=0.33$ (curve in yellow), $\beta _{o}=0.499$ (curve in violet). **b** Time series of $\widetilde{E}^{v}$ as $\gamma _{o}$ increases. Parameters: $\gamma _{o}=0.001$ (curve in blue), $\gamma _{o}=0.167$ (curve in orange), $\gamma _{o}=0.33$ (curve in yellow), $\gamma _{o}=0.499$ (curve in violet) (colour figure online)

An increase in $\beta _{o}$ or $\gamma _{o}$ implies an increase in externalities, and thus in strategic interactions between agents of the same generation. This phenomenon is profoundly different with respect to the one related to an increase of $\beta$ or $\gamma$. In fact, we recall that for the OLG structure, the decisions of the young are made when the decisions of the elderly have already been made. Therefore, a real strategic interaction between generations does not exist (an example of the dynamics generated as $\beta _{o}$ or $\gamma _{o}$ increases is given in Fig. 10).

**Fig. 11:**
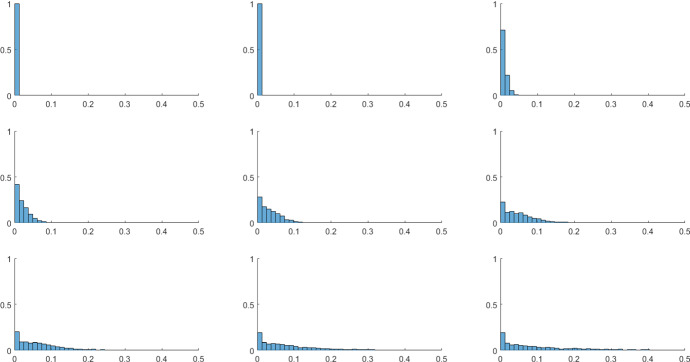
Distribution of $\Delta ^{\widetilde{E}^{v}}$ as *b* increases

Finally, a further factor that destabilizes the environmental dynamics is the speed of natural decay of the environment, *b* (see Fig. 11). An increase in *b* causes an effect in some way comparable to an increase in $\beta$. In fact, this increase implies that nature is less able to regenerate itself, and therefore, the negative externalities between generations generated by consumption are more important. This triggers a series of feedbacks already described in relation to $\beta$.

### Heterogeneous preferences

The aim of this subsection is to explore how the phenomena discussed so far change, when the homogeneity in the preferences of agents is removed. In order to do this, we study the simplest possible case, in which a neighborhood coincides with the entire grid. Therefore, for this numerical exercise, we assume $N=3$. In this case, we test the impact of heterogeneity by perturbing the preferences of only one of the cells. Starting from the situation of homogeneous agents and by increasing or decreasing the value of $\omega$ of a unique agent, said agent *p*, Monte Carlo simulations show that the variability of environmental dynamics is reduced (see Table 3).

**Table 3:** One heterogeneous preference for $N=3$ (2000 simulations)

Parameters	Convergent dynamics	Cycles ($\%$)
$\omega ^{v}=\omega ^{p}=\overline{\omega }$ for all 9 cells	443	$\simeq 78\%$
$\omega ^{v}=\overline{\omega }$ (in 8 cells), $\omega ^{p}<\omega ^{v}$	500	$75\%$
$\omega ^{v}=\overline{\omega }$ (in 8 cells), $\omega ^{p}>\omega ^{v}$	922	$\simeq 54\%$

**Table 4:** One heterogeneous preference for $N=10$ (2000 simulations)

Parameters	Convergent dynamics	Cycles ($\%$)
$\omega ^{v}=\omega ^{p}=\overline{\omega }$ for all 100 cells	443	$\simeq 78\%$
$\omega ^{v}=\overline{\omega }$ (in 99 cells), $\omega ^{p}<\omega ^{v}$	744	$\simeq 63\%$
$\omega ^{v}=\overline{\omega }$ (in 99 cells), $\omega ^{p}>\omega ^{v}$	998	$\simeq 50\%$

To understand this phenomenon, we must recall what explained for the homogeneous case. In that case, all agents take the same decision, causing a snowball effect, favorable to generate oscillatory dynamics. In this case instead, thanks to the interactions that agent *p* has with her neighbors, the presence of even only one agent who takes decisions structurally different ($\omega _{p}$ different from other $\omega$), reduces this phenomenon. As *N* increases, the phenomenon of stabilization tends to increase (see Table 4). To understand this result, we must consider that the agent *p*, or better, the generations of agents *p*, tend to stabilize the behaviors of the neighbors. The latter, in turn, become themselves different agents (for the allocative decisions they take), compared to their neighbors who do not have direct contact with *p*. This diffusion effect generated by agent *p*, tends to stabilize the dynamics on the entire grid.

To test the consistency of both (i) the stabilization phenomenon caused by the presence of heterogeneous preferences and (ii) the increase in the diffusion effect as *N* increases, we extend the analysis to the case where all the agents are heterogeneous. In particular, in the following simulations, we consider values of $\omega ^{v}$ on the grid extracted each time from a Beta-distribution, with probability density function $d(\omega ^{v};\rho ,\sigma )$ and where $\rho ,\sigma >0$ are the shape parameters of the distribution.^18^ At each simulation, both the shape parameters determining the Beta-distribution (from which each $\omega ^{v}$ is extracted) are sampled from a homogeneous distribution. Assuming a grid of dimension $N=3$, we notice that heterogeneous preferences on the whole grid induce a lower variability of the dynamics with respect to the case in which all agents have identical preferences. Indeed, Table 5 shows that in the case of heterogeneous $\omega ^{v}$ extracted from $d(\omega ^{v};\rho ,\sigma )$, the number of simulations whose variability $\Delta ^{\widetilde{E}^{v}}$ is close to zero increases.

**Table 5:** Heterogeneous preferences for $N=3$ (2000 simulations)

Parameters	Convergent dynamics	Cycles ($\%$)
$\omega ^{v}=\overline{\omega }$ for all 9 cells	443	$\simeq 78\%$
Each $\omega ^{v}$ is sampled from Beta-distribution	838	$\simeq 50\%$

This finding confirms what has already been shown above with a single perturbed cell. More specifically, the interaction among agents who have heterogeneous preferences favors a mitigation effect in the decisions of all agents in the long run. This in turn induces a partial reduction of oscillatory phenomena in the dynamics of the local environment on the grid, compared to what is observed when perfectly identical agents interact. This result shows a significant contrast to some results in the economic dynamics literature (e.g., Onozaki et al. 2003) in which heterogeneity is perceived as a vehicle for destabilizing the economic system.

**Fig. 12:**
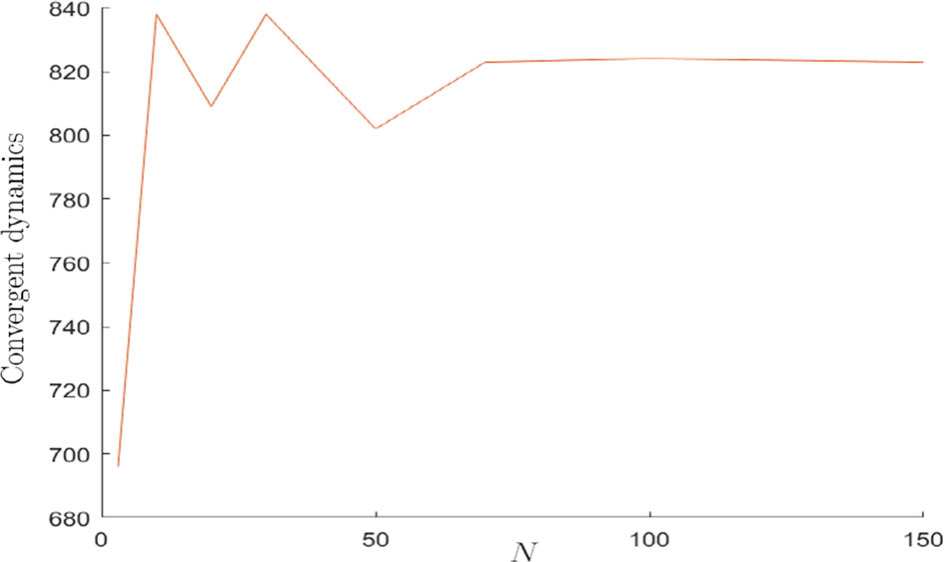
Variation in the number of convergent dynamics as the grid size *N* increases

Finally, we repeat the Monte Carlo simulations to test the role played by *N*, in this heterogeneous context. Starting from the grid size $N=3$, we notice that by increasing the grid size the number of convergent dynamics increases as well (see the left part of Fig. 12). This phenomenon can be interpreted as follows: considering those cases in which for low *N* a certain distribution of $\omega ^{v}$ on the grid did not allow a stabilization of the dynamics, we find that as the grid size increases, the same distribution of $\omega ^{v}$ produces the stabilization of the dynamics of $\widetilde{E}^{v}$ on *L*, due to the diffusion effect described before. The extent of the diffusion phenomenon tends to stabilize for sufficiently large *N* (see the right part of Fig. 12).

### The centralized case

Differently from what shown in the previous paragraphs (where a decentralized solution of the local environment problem is considered), in this paragraph we describe the result of assuming the existence of a short-lived social planner (see Pecchenino 1995) that maximizes the utility of the single cohort. Therefore, taking into account the network externalities created among the cells, the maximization problem is defined and follows:

$$\begin{aligned} \begin{aligned}&\underset{m_{t}^{v},s_{t}^{v}}{\text {max}} \ \ \sum _{v\in \,L}U^{v}(c_{t+1}^{v},E_{t+1}^{v}) \\&\text {subject to}\\&\ \ \ \ \ \ \ \ \ \ \ \ \ \ \ \ c_{t+1}^{v}=(1+r_{t+1}-\delta )s_{t}^{v} \ \ \ \ \ \ \ \ \ \forall v\in \,L \\&\ \ \ \ \ \ \ \ \ \ \ \ \ \ \ \ \ \ \ \ w_{t}^{v}=s_{t}^{v}+m_{t}^{v} \ \ \ \ \ \ \ \ \ \ \ \ \ \ \ \forall v\in \,L \\&E_{t+1}^{v}=(1-b)E_{t}^{v}+b\overline{E}^{v}+\bigg [\gamma ^{v}\,m_{t}^{v}+\sum _{l\in \,I_{v}} \gamma _{o}^{l}\,m_{t}^{l}\bigg ]-\bigg [\beta ^{v}\,c_{t}^{v}+\sum _{l\in \,I_{v}} \beta _{o}^{l}\,c_{t}^{l}\bigg ]\\&\ \ \ \ \ \ \ \ \ \ \ \ \ \ \ \ \ \ \ \ \ \ \ \ \ \ \ \ \ \ \ \ \ \ \ \ \ \ \ \ \ \ \ \ \ \ \ \ \ \ \ \ \ \ \ \ \ \ \ \ \ \ \ \ \ \ \ \ \ \ \ \ \ \ \ \ \ \ \ \ \ \ \ \ \ \ \ \ \ \ \ \ \forall v\in \,L\\&\ \ \ \ \ \ \ \ \ \ \ \ \ \ \ \ \ \ \ \ c_{t+1}^{v}>0, m_{t}^{v},s_{t}^{v}\ge \,0 \ \ \ \ \ \ \ \ \ \forall v\in \,L. \end{aligned} \end{aligned}$$

If the social planner internalizes the environmental problem, we notice that centralized decisions allow the achievement, on average, of higher environmental quality levels within *L*, compared with the levels obtained in the case of decentralized ones. Concerning the dynamics of the system, considering the values of parameters in Table 1, in the presence of centralized choices they converge all ($100\%$ of simulations performed) to stationary states $((\widetilde{E}^{v})^{*},k^{*})$, regardless of the size of the grid *N* and the assumption on $\omega$.

## Conclusions

In this article, by adapting the modeling framework introduced by John and Pecchenino (1994) to analyze the dynamic relationship between the environment and economic activity, we have focused on the analysis of the dynamics when a local dimension of the environment is considered. To this end, we have proposed an OLG agent-based model in which decisions are made in a decentralized form by a population of agents each of whom lives, with no possibility of migration, on a cell of a two-dimensional *lattice*. Agents make decisions according to the environmental level that is experienced in that cell, and consumption choices and expenditures to improve the environmental quality of their cells affect the environmental quality of neighboring cells through externalities that cross cell boundaries (local interactions). By analyzing the dynamics of the model, the main finding is that externalities among cells trigger strongly oscillatory dynamics, both in the case of homogeneous agents and in the presence of heterogeneous agents. The occurrence of cyclical dynamics in the local environmental quality is only partially mitigated by the presence of heterogeneity in individuals’ preferences. This means that differentiated behaviors among agents reduce the possibility of agents generating an ex-post situation on the network prone to induce strong feedbacks in agents’ *future* decisions. By studying the role of model parameters through sensitivity analysis, we have highlighted that the variables playing a destabilizing role in the dynamics are the level of the negative impact of consumption on the environment and the inability of the environment to regenerate at high rates, while the effectiveness of environmental spending and a strong general interest or disinterest in the environment are elements that favor the stabilization of the dynamics. Finally, when a centralized planner is introduced, the dynamics converge to stationary values regardless of the assumption on the heterogeneity of agents.

The model can be extended in several manners: first of all, it is possible to compare our model with the extensive literature focused on the definition of stable international environmental agreements (IEAs, hereafter). Recently, in this strand of the literature, Günther and Hellmann (2017) studied the conditions for the emergence and stability of environmental agreements and noticed that (i) the existence of stable agreements depends on the balance of network spillovers and that (ii) excessive asymmetries may lead to the instability of such agreements in the long run. These outcomes provide an interesting comparison to an extension of our model in which (contemporary) agents may create coalitions (IEAs). Indeed, an insight would be to observe how an overlapping generations model may react to coordination opportunities and consequently to study whether coalitions persist across generations (stable IEAs) or are subject to continuous (on/off) switches. Second, the modeling approach introduced in this article may be employed to study the decentralized decisions of agents on health in a context of deadly epidemic dynamics (e.g., SARS-CoV-2) affected by externalities arising from the behavior of agents and their neighbors (see Fernández et al. 2021).

## Figures and Tables

**Fig. 13:**
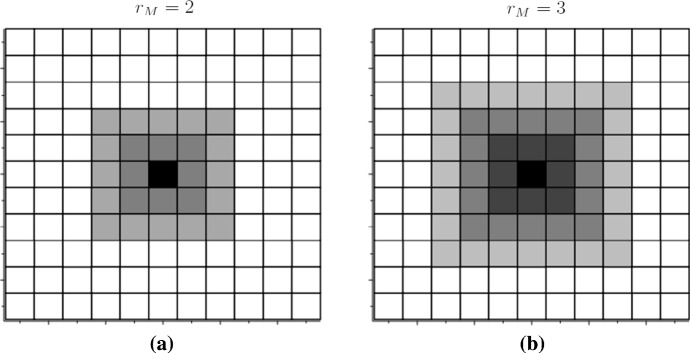
**a** An example of the Moore Neighborhood with $r_{M}=2$. **b** An example of the Moore Neighborhood with $r_{M}=3$. In both examples, the black cell identifies the agent *v* and the gray ones her neighbors. The different shades of gray recall the different impact that agents at a different radius have on the cell *v*

## Appendix 1

When the interior solution of the optimization problem applies in every cell *v*, we notice that the map describing the dynamics of the system, considering equations (12)–(13)–(14)–(15) introduced in the body text, can be written as a system of second order difference equations in the variables $E^{e,v}$, with *v* belonging to *L*. Then, the system reads as

$$\begin{aligned} E_{t+1}^{e,v}&=(1-b)\left[ E_{t+1}^{e,v}+\gamma _{o}\sum _{l\in I_{v} }\left( A(1-\alpha )\left[ \frac{1}{N^{2}}\sum _{v=1}^{N^{2}}\frac{E_{t} ^{e,v}}{\gamma ^{v}\eta ^{v}}\right] ^{\alpha }-\frac{E_{t+1}^{e,l}}{\gamma ^{v}\eta ^{v}}\right) \right] +b\overline{E}\\&\quad -\left\{ \sum _{l\in I_{v}}\left( \beta _{o}\left[ 1+\alpha A\left[ \frac{1}{N^{2}}\sum _{v=1}^{N^{2}}\frac{E_{t}^{e,v}}{\gamma ^{v}\eta ^{v} }\right] ^{\alpha -1}-\delta \right] \frac{E_{t}^{e,l}}{\gamma ^{v}\eta ^{v} }\right) \right. \\&\quad \left. +\beta \left[ 1+\alpha A\left[ \frac{1}{N^{2}}\sum _{v=1}^{N^{2} }\frac{E_{t}^{e,v}}{\gamma ^{v}\eta ^{v}}\right] ^{\alpha -1}-\delta \right] \frac{E_{t}^{e,v}}{\gamma ^{v}\eta ^{v}}\right\} \\&\quad +\left\{ b\gamma _{o}\sum _{l\in I_{v}}\left[ A(1-\alpha )\left[ \frac{1}{N^{2}}\sum _{v=1}^{N^{2}}\frac{E_{t-1}^{e,v}}{\gamma ^{v}\eta ^{v}}\right] ^{\alpha }-\frac{E_{t}^{e,l}}{\gamma ^{v}\eta ^{v}}\right] \right. \\&\quad \left. +\gamma \left[ A(1-\alpha )\left[ \frac{1}{N^{2}}\sum _{v=1}^{N^{2}}\frac{E_{t}^{e,v}}{\gamma ^{v}\eta ^{v}}\right] ^{\alpha }-\frac{E_{t+1}^{e,v}}{\gamma ^{v}\eta ^{v}}\right] \right\} \end{aligned}$$

where $\beta _{o}^{l}=\beta _{o}$, $\gamma _{o}^{l}=\gamma _{o}$, $\beta ^{v}=\beta$ and $\gamma ^{v}=\gamma$. This system can be written also as the following $N^{2}+2$ dimensions dynamic system in the variables $E_{t}^{e}$, $k_{t}$ and $p_{t}$ (where $p_{t}=k_{t-1}$)

34 $$\begin{aligned} F: {\left\{ \begin{array}{ll} E_{t+1}^{e,v} &{}=(1-b)\left[ E_{t+1}^{e,v}+\gamma _{o}\sum _{l\in I_{v}}\left( A(1-\alpha )k_{t}^{\alpha }-\frac{E_{t+1}^{e,l}}{\gamma \eta }\right) \right] +b\overline{E}\\ &{}\quad -\left\{ \sum _{l\in I_{v}}\left( \beta _{o}\left[ 1+\alpha Ak_{t}^{\alpha -1}-\delta \right] \frac{E_{t}^{e,l}}{\gamma \eta }\right) +\beta \left[ 1+\alpha Ak_{t}^{\alpha -1}-\delta \right] \frac{E_{t}^{e,v} }{\gamma \eta }\right\} \\ &{}\quad +\left\{ b\gamma _{o}\sum _{l\in I_{v}}\left[ A(1-\alpha )p_{t}^{\alpha } -\frac{E_{t}^{e,l}}{\gamma ^{v}\eta ^{v}}\right] +\gamma \left[ A(1-\alpha )k_{t}^{\alpha }-\frac{E_{t+1}^{e,v}}{\gamma \eta ^{v}}\right] \right\} \\ p_{t+1} &{}=k_{t}\\ k_{t+1} &{}=\frac{1}{\gamma \eta N^{2}}\sum _{v=1}^{N^{2}}E_{t+1}^{e,v}. \end{array}\right. } \end{aligned}$$

We notice that in *F* the $N^{2}$ variables $E_{t+1}^{e,v}$, with *v* belonging to *L*, appear on both the sides of the expression. Given the linearity and the assumptions on the parameters, it is possible to write the dynamic system in an explicit form, and therefore, we have

$$\begin{aligned} F: {\left\{ \begin{array}{ll} E_{t+1}^{e,v} &{} =g(\mathbf {E}_{t}^{e},k_{t},p_{t}),\text { }v\in \,L\\ p_{t+1} &{} =k_{t}\\ k_{t+1} &{} =\frac{1}{\gamma \eta N^{2}}\sum _{v=1}^{N^{2}}g(\mathbf {E}_{t}^{e} ,k_{t},p_{t}) \end{array}\right. } \end{aligned}$$

where $\mathbf {E}_{t}^{e}$ is the vector collecting the values of $E_{t}^{e}$ on all the cells of *L*. Assuming that $\overline{E}=0$, we can derive the stationary state of the system in closed form

$$\begin{aligned} \left( E^{e,v}\right) ^{*}&=\left[ \frac{\beta \delta -8(1-\delta )\beta _{o}-b\eta \gamma -\beta -\gamma -8\gamma _{o}}{((\beta +8\beta _{o} +\gamma +8\gamma _{o})\alpha -\gamma -8\gamma _{o})A}\right] ^{1/(\alpha -1)} \gamma \eta \\ p^{*}&=k^{*}=\frac{\left( E^{e,v}\right) ^{*}}{\gamma \eta }. \end{aligned}$$

This stationary state exists when the expression $\frac{\beta \delta -8(1-\delta )\beta _{o}-b\eta \gamma -\beta -\gamma -8\gamma _{o}}{((\beta +8\beta _{o} +\gamma +8\gamma _{o})\alpha -\gamma -8\gamma _{o})A}$ exists and it is positive. Under the hypothesis of identical initial condition for (*E*, *k*, *p*), the dynamics of *E* in the system evolve on the subset

$$\begin{aligned} E_{t}^{e,i}=E_{t}^{e,j} \end{aligned}$$

with $i,j=1\ldots N^{2}$. In this case, the dynamics of every cell can be described by the two-dimensional map

35 $$\begin{aligned} \hat{F}: {\left\{ \begin{array}{ll} E_{t+1}^{e} &{}=\frac{1}{\gamma \, ( 1+\eta ) } ( 8\,A\gamma _{o}\, \gamma \,\eta \, (1-\alpha ) \big ( {\frac{{p_{t}}}{ \gamma \,\eta }} \big ) ^{\alpha }+A{\gamma }^{2}\eta \, ( 1-\alpha ) \big ( {\frac{{E_{t}^{e}}}{\gamma \,\eta }} \big ) ^{\alpha }+\\ &{}( \eta \gamma ( 1-b )- ( 1+A\alpha \, \big ( {\frac{{ E_{t}^{e}}}{\gamma \,\eta }} \big ) ^{\alpha -1}-\delta ) ( \beta +8\,\beta _{o} ) ) { E_{t}^{e}}-8\,\gamma _{o}{ p_{t}} ) \\ p_{t+1} &{}=E_{t}. \end{array}\right. } \end{aligned}$$

Considering the expressions in the map $\hat{H}$, the Jacobian matrix evaluated at the stationary state $((E^{e})^{*},p^{*})$ reads as

$$\begin{aligned} W((E^{e})^{*},p^{*})={ \left[ \begin{array}{cc} W_{11}^{*} &{}\quad W_{12}^{*} \\ 1 &{}\quad 0 \end{array} \right] }, \end{aligned}$$

where $W_{11}^{*}=\frac{\partial \,E_{t+1}^{e}}{\partial \,E_{t}^{e}}((E^{e})^{*},p^{*})$ and $W_{12}^{*}=\frac{\partial \,E_{t+1}^{e}}{\partial \,p_{t}}((E^{e})^{*},p^{*})$. Specifically, we have

$$\begin{aligned} W_{11}^{*}&=\frac{1}{( 1+\eta ) ( ( \beta +8\,\beta _{o}+8\, \gamma _{o}+\gamma ) \alpha -8\,\gamma _{o}-\gamma ) \gamma }\big [( \beta +8\,\beta _{o}+\gamma )(( b\eta + 1) \gamma +8\,\gamma _{o}\\&\quad +( -8\,\delta +8) \beta _{o}+( -\delta +1) \beta ) {\alpha }^{2}+(( -1+( -2\,b+1) \eta ) {\gamma }^{2}\\&\quad +(( 16\,\delta -16+ ( -8\,b+8 ) \eta ) \beta _{o}+ ( 2\, \delta -2+ ( 1-b ) \eta ) \beta -8\, ( 1+\eta \, ( -1+b )) \gamma _{o} ) \gamma \\&\quad +( \beta +8 \,\beta _{o}) ( \beta +8\,\beta _{o}+8\,\gamma _{o})( \delta -1 ) ) \alpha +8\, ( \gamma _{o}\\&\quad +\frac{\gamma }{8}) ( \eta \,( -1+b ) \gamma - ( \delta -1 ) ( \beta +8\,\beta _{o} ) ) \big ], \\ W_{12}^{*}&=\frac{8}{( 1+\eta )(( \beta +8\, \beta _{o}+8\,\gamma _{o}+\gamma ) \alpha -8\,\gamma _{o}-\gamma ) }\big [(( b\eta \,\gamma -\beta \,\delta -8\,\beta _{o}\, \delta +\beta \\&\quad +8\,\beta _{o}+8\,\gamma _{o}+\gamma ) {\alpha }^{2}\\&\quad +( -b\eta \,\gamma +\beta \,\delta +8\,\beta _{o}\,\delta -2\,\beta -16\, \beta _{o}-16\,\gamma _{o}-2\,\gamma ) \alpha +8\,\gamma _{o}+\gamma ) \gamma _{o}\big ]. \end{aligned}$$

The local stability of $((E^{e})^{*},p^{*})$ is described by the so-called Jury conditions:

$$\begin{aligned}&1-Tr(W^{*})+Det(W^{*})>0 \\&1+Tr(W^{*})+Det(W^{*})>0 \\&1-Det(JJ^{*})>0 \end{aligned}$$

where $Tr(W^{*})=W_{11}^{*}$ and $Det(W^{*})=-W_{12}^{*}$. Since it is possible to violate, with an appropriate parametric configuration, both the second and third Jury conditions, we can notice that when the steady state is locally stable, it can lose its stability either through a Flip bifurcation (when $1+Tr(W^{*})+Det(W^{*})=0$) or a Neimark–Sacker bifurcation (when $Det(W^{*})=1$).

## Appendix 2

In the main body of the text, the case of a Moore neighborhood $I_{v}$ with a unit radius $r_{M}=1$ has been considered. What happens if we consider that the environmental quality in a single cell, $E_{t}^{v}$, is also affected by the decisions made in cells with a higher distance from *v*? In this case, we redefine the Moore Neighborhood of the cell *v* as $N_{v}=\{l:|v-l|\le \,r_{M}\}$ (see Fig. 13), with $r_{M}\ge \,1$, and the dynamics of $E_{t}^{v}$ would be described by the equation:

36 $$\begin{aligned} E_{t+1}^{v}=(1-b)E_{t}^{v}+b\overline{E}^{v}+\gamma ^{v} m_{t}^{v}+\sum _{l\in \,N_{v}} \bigg (\frac{\gamma _{o}^{l}}{r_{M}^{l}}\bigg )\,m_{t}^{l}-\beta ^{v} c_{t}^{v}-\sum _{l\in \,N_{v}} \bigg (\frac{\beta _{o}^{l}}{r_{M}^{l}}\bigg )\,c_{t}^{l}\quad \end{aligned}$$

where $r_{M}^{l}\le \,r_{M}$ weights the impact of the decisions made by agents around the agent *v* in the neighborhood. Then, following the assumption made in the description of the model ($\beta _{o}^{l}<\beta ^{v}$, $\gamma _{o}^{l}<\gamma ^{v}$), the higher is the distance between any agent *l* and *v*, the less is the impact of *l*’s choices on the *v*’s ones.

Recalling all the assumptions made at the beginning of Sect. 4 and performing 2000 replications of the model with $N=10$, we test the role of the radius $r_{M}$, that is the impact of considering larger (symmetric) Moore neighborhood on *L*. The results in Table 6 allow us to observe that, as the radius assumed in defining the neighborhoods $N_{v}$ on *L* increases, the dynamics of the system tend (statistically) to converge more and more toward the stationary equilibrium.

**Table 6:** The role of the radius $r_{M}$

Parameters	Convergent dynamics	$\%$ Cycles
$r_{M}=1$	438	$78\%$
$r_{M}=2$	791	$60\%$
$r_{M}=3$	1141	$43\%$
$r_{M}=4$	1612	$19\%$
$r_{M}=5$	1875	$6\%$

In this case, we can notice that the impact of other individuals’ actions in defining the environment of the single cell becomes increasingly important in defining the long run dynamics of the cells. Indeed, considering a higher $r_{M}$, the free riding phenomenon of agents in the grid increases, and it induces, as common in the literature, the convergence of environmental dynamics toward low environmental levels (due to the low value of individuals contributing to the environmental maintenance). Moreover, as $r_{M}$ approaches the value $r_{M}^{max}$ (where all the grid is considered as a unique neighborhood) the model becomes similar to Naimzada and Sodini (2010).

## Footnotes

1 The result is in line also with the Green Solow model (Brock and Taylor 2010), where the authors develop the theme of the interactions between the environment and economic growth using a Solow environmental-augmented model.

2 This phenomenon is called maladaptation. Barnett and O’Neill (2010) introduce the definition of maladaptation by describing that series of actions aimed at apparently reducing the environmental impact but ending up generating further negative effects on the environment.

3 At $t=0$ there exists a unique generation of young agents.

4 The movement of agents, and in particular their migration, due to environmental issues is a topic that has recently been the object of many studies, including Marchiori and Schumacher (2011) and has also been considered in several ABMs, such as the well-known *Sugarscape Model* proposed by Epstein and Axtell (1996). However, in the present model, in order to avoid an excessively complicated analytical structure, we assume that the life of the agent unfolds entirely in the cell in which she was born.

5 For the sake of clarity, the term $E_{t}^{v}$ could be interpreted as (i) the index describing the current level of environmental amenities or as (ii) the stock of the free access environmental good at *t*.

6 We notice that in the present model, externalities related to consumption (and thus production) only affect the environmental good. Recent events (the SARS-CoV-2 epidemic) have shown that externalities could also have a strong impact on life expectancy, here considered as exogenously given.

7 At $t=0$, i.e., at the period in which the first generation of individuals born, agents expect that there are no externalities arising from the behavior of others.

8 Since *(i)* the consumption of agents in old age is generated by the saving defined at time $t-1$ (i.e., an observable historical data at time *t*) and *(ii)* the real interest rate is given for agents, we assume that the consumption of old individuals in *t* is known to young agents.

9 The equation in (11) shows a relationship between individual savings (and therefore capital) and expected local environmental quality. This relationship is similar to the one obtained in Zhang (1999) and Naimzada and Sodini (2010), but it is based on expectations, which may be different from the *actual* environmental values because agents are not able to perfectly foresee the choices of their neighbors.

10 To the best of our knowledge, there are no estimations on *b* and so we will need to consider in the model analysis *b* varying in its entire spectrum (0, 1).

11 This value is calculated by assuming an annual depreciation rate equals to 0.05 and that each period lasts 25 years (see also Barro and Sala-i Martin 2004).

12 For completeness, the official source is: University of Groningen and University of California, Davis, Capital Stock at Constant National Prices for Italy [RKNANPITA666NRUG], retrieved from FRED, Federal Reserve Bank of St. Louis; https://fred.stlouisfed.org/series/RKNANPITA666NRUG.

13 The use of scaling factors to classify the dynamical properties of an economic model is a widespread technique. See for example Grandmont et al. (1998).

14 Through a very large number of simulations, we can state that increasing the sample size does not change the results of the analysis performed.

15 With this specification, we actually consider the evolution of the model since the beginning of the productive human activity. Naturally, the first iterations are characterized by an extremely low or null level of environmental expenditures.

16 For the coefficient of variation, there can be some numerical problems related to values close to 0 of the denominator. Some techniques to reduce rounding problems need to be implemented.

17 In this case, as explained in the introductory part of the section the results do not depend on *N*.

18 To avoid having $\mu (\omega ^{v})=0.5$ and $Var(\omega ^{v})=1/12$ in all simulations, we have preferred the Beta-distribution over the Uniform distribution. The Beta-distribution allows to change both mean and variance of the distribution.
